# Controversies in the treatment of RAS wild-type metastatic colorectal cancer

**DOI:** 10.1007/s12094-020-02475-8

**Published:** 2020-08-13

**Authors:** R. Vera, M. Salgado, M. J. Safont, J. Gallego, E. González, E. Élez, E. Aranda

**Affiliations:** 1 Medical Oncology Department, Complejo Hospitalario de Navarra, Pamplona, Spain; 2 Medical Oncology Department, Complejo Hospitalario Universitario de Ourense, Ourense, Spain; 3 Medical Oncology Department, Hospital General Universitario de Valencia, Valencia, Spain; 4 Medical Oncology Department, Hospital General Universitario de Elche, Alicante, Spain; 5 Medical Oncology Department, Hospital Universitario Virgen de las Nieves, Granada, Spain; 6 Vall d’Hebron Institute of Oncology, Barcelona, Spain; 7 Medical Oncology Department, Maimonides Institute of Biomedical Research (IMIBIC), Hospital Reina Sofía, University of Córdoba, Av. Menendez Pidal, s/n, 14004 Córdoba, Spain

**Keywords:** Metastatic colorectal cancer, *RAS* wild-type, Treatment patterns, Primary tumor sidedness, Maintenance, Liquid biopsy, Rechallenge, Delphi

## Abstract

**Objective:**

To provide guidance for the management of *RAS* wild-type (wt) metastatic colorectal cancer (mCRC) in daily practice.

**Methods:**

Nominal group and Delphi techniques were used. A steering committee of seven experts analyzed the current management of *RAS* wt mCRC, through which they identified controversies, critically analyzed the available evidence, and formulated several guiding statements for clinicians. Subsequently, a group of 30 experts (the expert panel) was selected to test agreement with the statements, through two Delphi rounds. The following response categories were established in both rounds: 1 = totally agree, 2 = basically agree, 3 = basically disagree, 4 = totally disagree. Agreement was defined if ≥ 75% of answers were in categories 1 and 2 (consensus with the agreement) or 3 and 4 (consensus with the disagreement).

**Results:**

Overall, 71 statements were proposed, which incorporated the following areas: (1) overarching principles; (2) tumor location; (3) triplets; (4) maintenance; (5) second-line and beyond treatments; (6) Rechallenge and liquid biopsy. After the two Delphi rounds, only six statements maintained a lack of clear consensus.

**Conclusions:**

This document aims to describe the expert’s attitude when dealing with several common clinical questions regarding patients with *RAS* wt mCRC.

**Electronic supplementary material:**

The online version of this article (10.1007/s12094-020-02475-8) contains supplementary material, which is available to authorized users.

## Introduction

Colorectal cancer (CRC) is the fourth most commonly diagnosed malignancy and the second-leading cause of global cancer-related deaths [1]. Approximately 20–25% of patients exhibit metastatic disease (mCRC) at disease onset and 50% of patients will eventually develop metastases [2].

The prognosis of mCRC has dramatically improved in recent decades, due to a range of factors, including improvements in treatment strategies and new biological agents [3–5].

Another factor that has contributed to this improvement is biomarker-based patient selection. *RAS* mutations have been associated with a lack of response to anti-epidermal growth factor receptor (EGFR) monoclonal antibody therapies, which are used in mCRC treatment [6, 7]. Based on these data, guidelines from the European Society for Medical Oncology (ESMO) note that expanded *RAS* analyses should be conducted on all patients at the time of diagnosis of mCRC, as well as on all patients that are eligible or being considered for anti-EGFR therapy [8]. However, this requirement has increased the need for more information about clinical and tumor characteristics based on *RAS* mutational status. For example, in *RAS* wild-type (wt) mCRC patients, published data suggest that primary tumor location might have a predictive effect, depending on the treatment applied [9, 10].

Consequently, oncologists may face different questions in daily practice, when considering the best treatment option to manage *RAS* wt mCRC patients. Therefore, the aim of this consensus document was to provide a guide to managing *RAS* wt mCRC patients, focusing on those areas that might generate clinical questions or controversies.

### Methodology

Nominal group and Delphi techniques were used, and a comprehensive narrative review supported the statements.

#### Expert panel selection and clinical statement generation

A steering committee of seven experts on mCRC was established, who were responsible of: (1) the selection of the expert panel; (2) identification of current relevant clinical questions and controversies in the field; (3) generation of statements. These statements were subsequently organized into six main sections: (a) general aspects; (b) tumor sidedness; (c) chemotherapy (CT) triplets; (d) maintenance; (e) second-line and beyond treatments; (f) rechallenge and liquid biopsy; (4) Interpretation of the results from the Delphi rounds; (5) Final edition of the document.

The expert panel comprised 30 experts that were selected according to the following criteria. Experts must be medical oncologists, specialize in mCRC, have clinical experience ≥ 8 years or ≥ 5 publications, and be members of the Sociedad Española de Oncología Médica (SEOM) or Grupo Español de Tratamiento de Tumores Digestivos (TTD).

#### Delphi process

The expert panel completed two Delphi rounds using an online platform. After each round, a facilitator provided an anonymous summary of the experts’ forecasts as well as the individual responses of each expert from the previous round. In the first round, the panelists voted using the following options: 1 = totally agree, 2 = basically agree, 3 = basically disagree, 4 = totally disagree. Consensus was defined if there were ≥ 75% of answers in categories 1 or 2 or 3 or 4. Any statement that reached consensus in this round did not proceed to a second round. The rest of the statements were analyzed by the steering committee, who reformulated the statement or maintained the original statement for the next Delphi round. In the second round, votes for statements employed the same categories. However, in this stage, “consensus” was defined if ≥ 75% of responses in categories 1 and 2 (sum of the responses of both categories), consensus in the agreement, or in categories 3 and 4 (sum of the responses of both categories), consensus in the disagreement. When the rate of sum of responses in categories 1 and 2 or 3 and 4 was 60%–75%, this was considered “majority” and when it was < 60%, this was considered “dissent.” Finally, if this rate was 100%, this was considered “unanimous.”

## Results

### Delphi process

Overall, 71 statements were generated. After the first Delphi round, 13 reached consensus and three were rephrased. After the second round, the grade of the agreement was 8 “unanimous,” 47 “consensus,” 10 “majority,” and 6 “dissent” statements. Please see the supplementary material (tables a to f) for more details of the Delphi results. Tables 1, 2, 3, 4, 5 and 6 summarize the main conclusions of the sections described below.

**Table 1:** Main conclusions of the overarching principles section

1	In mCRC, to select a treatment appropriately, the assessment of *RAS/BRAF* mutational status and the MSI is strongly recommended
2	In mCRC patients, I consider the best treatment option in first-line the combination of CT and biologic therapy in fit patients
3	In mCRC patients, I consider a multidisciplinary team necessary for the treatment of patients with mCRC

*wt* wild-type, *mCRC* metastatic colorectal cancer, *CT* chemotherapy, *MSI* microsatellite instability

**Table 2:** Main conclusions of the primary tumour sidedness section

#	Conclusions
1	In *RAS* wt mCRC patients, primary tumour location (right or left colon) is a factor to be taken into account when selecting a treatment. However, the molecular characteristics of the primary tumour are the most important factor
2	Treatment goal (response vs. survival) in another factor to be considered when selecting a treatment
3	In relation to the first-line treatment in *RAS* wt mCRC patients:
	In right-sided primary tumors, when treatment goal is response, and in the absence of contraindications, the preferred treatments are CT triplets + bevacizumab
	In right-sided primary tumors, when treatment goal is survival, and in the absence contraindications, the preferred treatments are CT doublets + bevacizumab
	In left-sided primary tumors, when treatment goal is response, and in the absence contraindications, the preferred treatments are Ct doublets + anti-EGFR therapy
	In left-sided primary tumors, when treatment goal is survival, and in the absence contraindications, the preferred treatments are Ct doublets + anti-EGFR therapy

*wt* wild-type, *mCRC* metastatic colorectal cancer, *CT* chemotherapy, *EGFR* epidermal growth factor receptor

**Table 3:** Main conclusions of the triplets section

#	Conclusions
1	In general, in *RAS* wt mCRC patients, the use of first-line CT triplets + targeted therapy is influenced by:
	Treatment goal
	Hard-to-handle toxicity
	The impact (limitation) of this strategy on second-line treatment options
2	It can be considered in first-line treatment:
	CT triplets + antiangiogenic therapy in right-sided primary tumors potentially resectables
	In left-sided primary tumors potentially resectables, CT doublets + anti-EGFR therapy is more appropriate than CT triples + anti-EGFR or antiangiogenic therapy
	In *BRAF* mt mCRC patients, CT triplet + antiangiogenic drugs is the preferred option
3	It can be considered in second-line treatment:
	The use of anti-EGFR drugs after progression to a first-line CT triplet + anti-angiogenic drugs
	The use of CT + anti-angiogenic drugs after progression to a first-line CT triplet + anti-EGFR therapy

*wt* wild-type, *mCRC* metastatic colorectal cancer, *CT* chemotherapy, *EGFR* epidermal growth factor receptor

**Table 4:** Main conclusions of the maintenance section

#	Conclusions
1	Maintenance in the first-line treatment of unresectable mCRC patients is a treatment standard in clinical practice
2	Complete cessation of first-line CT and biologicals (once the maximum response is achieved), and intermittent treatment with pre-established periods of rest (for CT and biologicals), are not a preferred alternative to maintenance
3	Induction first-line treatment of unresectable mCRC patients should be maintained until the maximum response is achieved (in the absence of contraindications), adapting the treatment scheme and evaluations to patients and tumor features and local organization
4	In unresectable mCRC patients with first-line CT + bevacizumab treatment, it would be advisable to maintain treatment with fluoropyrimidines + bevacizumab until disease progression
5	In unresectable *RAS* wt mCRC patients with first-line CT + anti-EGFR therapy, EGFR inhibitors (in the absence of unmanageable toxicity) should be maintained until disease progression
6	Anti-EGFR or antiangiogenic monotherapy is not recommended as maintenance therapy in unresectable mCRC
7	At disease progression after maintenance treatment, the first option is the reintroduction of initial induction therapy (in the absence of relevant residual toxicity)

*wt* wild-type, *mCRC* metastatic colorectal cancer, *CT* chemotherapy, *EGFR* epidermal growth factor receptor

**Table 5:** Main conclusions of second-line treatment and beyond section

#	Conclusions
1	The combination of CT + targeted treatment should be a part of the second-line treatment in most of *RAS* wt fit mCRC patients, and the oncology treatment is a reality in the third-line treatment and beyond
2	The response rate for possible resectability or for symptom control is a treatment goal to be considered when selecting treatment in second line
3	Primary tumor sidedness does not influence the selection of treatment in the second line
4	In *RAS* wt mCRC who have received first-line anti-EGFR therapy, the preferred treatment in second line is an antiangiogenic drug
5	In *RAS* wt mCRC who have not received anti-EGFR treatment in first or second line, the preferred option in the third line and beyond is anti-EGFR therapy ± CT
6	Toxicity is the most important factor in the selection of treatment mCRC patients beyond second line
7	It would be important to have in clinical practice MSI and *HER2* data for the treatment decision making in the third line and beyond

*wt* wild-type, *mCRC* metastatic colorectal cancer, *CT* chemotherapy, *EGFR* epidermal growth factor receptor, *MSI* microsatellite instability

**Table 6:** Main conclusions of the rechallenge and liquid biopsy section

#	Conclusions
1	In *RAS* wt mCRC patients the assessment of *RAS* mutational status (exons 2, 3, 4 of *KRAS* and *NRAS*) using liquid biopsy is a STANDARD alternative comparable to paraffin
2	The detection sensitivity of the liquid biopsy technique is a decisive factor in the decision-making process of *RAS* wt mCRC patients
3	Currently (in the absence of more robust data), rechallenge with anti-EGFR therapies is generally not considered to be a comparable alternative to approved standard therapies in patients who have received all therapies considered standard in *RAS* wt disease. But if rechallenge *is* considered, liquid biopsy should be used to make this decision
4	Reintroduction of anti-EGFR therapies could be a comparable alternative to approved standard therapies in patients who have received all therapies considered standard in *RAS* wt disease
5	It is important to define the exact cut-off for clinically relevant *RAS*-mutant allele frequencies in liquid biopsy, i.e. the MAF below which the patient is considered to still benefit from anti-EGFR treatment

*wt* wild-type, *mCRC* metastatic colorectal cancer, *CT* chemotherapy, *EGFR* epidermal growth factor receptor, *MAF* mutant allele fraction

### Overarching principles (Table 1)

The experts agreed (100%, “unanimous”) the following statements regarding mCRC patients. First, *RAS/BRAF* mutational status and the microsatellite instability (MSI) assessment are strongly recommended to appropriately select a treatment. Second, the best first-line treatment option is the combination of CT and biologic therapy, in fit patients. Third, care should be provided by a multidisciplinary team. These Delphi results are depicted in Table 1 of the supplementary material.

### Primary tumor sidedness (Table 2)

Experts considered (90% agreement, “consensus”) that primary tumor location should be taken into account in first-line treatment selection for *RAS* wt mCRC. However, the differences described between the right and left colon may determine treatment approaches [9, 11–13]. The treatment goal was also considered relevant, but the molecular profile was deemed the most crucial factor for treatment selection, as expounded by the guidelines [14].

In patients with right-sided mCRC, when the treatment goal was response, and in the absence of contraindications, the panelists preferred first-line CT triplet + bevacizumab (80% agreement, “consensus”). Sub-analyses in right-sided *RAS*-*BRAF wt* mCRC showed no benefit in response rates with FOLFOXIRI + bevacizumab versus FOLFOX + bevacizumab (81.3% vs. 66.7%; *p* = 0.584) [15]. Propensity score-match analyses indicated that adding bevacizumab to FOLFOXIRI provided significant benefits in terms of progression-free survival (PFS) and overall survival (OS) (HR = 0.73; 95% CI 0.55–0.97) without increasing the response rate (HR = 1.29; 95% CI 0.81–2.05) [16].

CT doublet + anti-EGFR was selected by 60% of experts for patients with right-sided mCRC when looking for a response. In the PRIME study, there were no differences in the response rates between FOLFOX + panitumumab (42.1%) and FOLFOX (34.8%) [17], while in the PEAK trial, the corresponding response rates were 63.6% for panitumumab + mFOLFOX versus 50% for bevacizumab + mFOLFOX (OR = 1.75; 95% CI 0.36–8.39) [17]. Data from the FIRE-3 study did not reveal any significant differences between FOLFIRI + bevacizumab versus FOLFIRI + cetuximab in the response rates (OR = 1.11; 95% CI 0.48–2.59) [18]. Similar results were observed in the Phase III CALGB/SWOG 80405 trial [19].

With regards to CT triplet + anti-EGFR, right-sided *RAS* wt mCRC patients in the Phase II VOLFI trial exhibited higher response rates with mFOLFOXIRI + panitumumab versus FOLFOXIRI (70.0% vs. 37.5%; *p* = 0.345) [20]. Several Phase II randomized controlled trials (RCTs) with cetuximab, without specific sub-analyses and based on tumor location, have shown benefits in the response rates [21, 22]. A meta-analysis involving patients with right-sided *RAS* wt mCRC revealed that CT + anti-EGFR achieved higher response rates than CT + bevacizumab or CT alone (OR = 0.56; 95% CI 0.40–0.79 and OR = 0.56; 95% CI 0.36–0.87, respectively) [9]. However, it must be noted that these estimates are based on relatively small sample sizes.

In right-sided mCRC, when the treatment goal was survival, the panelists preferred first-line CT doublets + bevacizumab (93% agreement, “consensus”). CT triplets + bevacizumab was chosen by 70% of experts (“majority”). In the TRIBE trial, the median OS in *RAS* and *BRAF* wt right-sided mCRC patients was 31.5 months for FOLFOXIRI + bevacizumab versus 22.3 months for FOLFIRI + bevacizumab (*p* = 0.165), while the median PFS was 13.4 versus 10.1 months (*p* = 0.292) [15]. Adverse events were significantly higher in the CT-triplet group [14]. Furthermore, a meta-analysis suggests that right-sided *RAS* wt mCRC patients achieve higher OS and PFS with CT, with or without bevacizumab, compared to CT + anti-EGFR [9]. However, these data are similarly based on small sample sizes.

Panelists did not recommend CT doublets (87%, “consensus”) or triplets (93%, “consensus”) + anti-EGFR in right-sided *RAS* wt mCRC when the treatment goal was survival. In the PRIME study, patients with these features depicted a median OS of 11.1 months with FOLFOX + panitumumab, compared to 15.4 months with FOLFOX (HR = 0.87; 95% CI 0.55–1.37; *p* = 0.539). Median PFS was 7.5 months versus 7 months (HR = 0.80; 95% CI 0.51–1.26; *p* = 0.328) [17]. The PEAK study [17], as well as the FIRE-3 and CALGB/SWOG 80405 studies both of which concerned cetuximab, all reported similar results [18, 19]. The above-mentioned meta-analysis suggested better OS and PFS for CT + bevacizumab, compared to CT + anti-EGFR [9]. The VOLFI trial demonstrated 6.3 months PFS for CT triplet + panitumumab versus 8.5 months for CT triplet (*p* = 0.338) [20]. Moreover, the panelists recognized the toxicity of CT + anti-EGFR, while evidence from another Phase II trial for the triplet could limit these therapeutic options [20, 23].

In patients with left-sided *RAS* wt mCRC, 60% of the experts (“majority”) considered that selecting a treatment should consider the treatment goal and tumor’s molecular profile. Nevertheless, with regards to treatment goal, the experts preferred (93% agreement, “consensus”) CT doublet + anti-EGFR for first-line treatment. Response rates in the PRIME study of this population were higher with FOLFOX + panitumumab than with FOLFOX (OR = 1.91; 95% CI 1.18–3.07) [17]. Similarly, median OS and PFS were higher for CT doublet + panitumumab, at 32.5 months versus 23.6 months (*p* < 0.001), and 12.9 months versus 9.3 months (*p* = 0.002), respectively. In the PEAK study, the median OS favored CT + panitumumab over CT + bevacizumab (HR = 0.77; 95% CI 0.46–1.28) [17]. Additionally, in the FIRE-3 study, the response rate for FOLFIRI + cetuximab was higher than for FOLFIRI + bevacizumab, at 61.7% versus 68.8% (HR = 1.37; 95% CI 0.85–2.19) [18]. In this and the CALGB/SWOG 80405 studies, OS was significantly higher for cetuximab [18, 19].

When the treatment goal was the response, CT triplets + anti-EGFR or bevacizumab were not the preferred options (53% and 57% of experts, respectively). The response rate in left-sided *RAS* wt mCRC patients from the VOLFI trial was higher with mFOLFOXIRI + panitumumab than with FOLFOXIRI (OR = 4.52; 95% CI 1.30–15.72) [20]. Other Phase II studies have analyzed CT triplet + cetuximab, which suggest clinical benefit [21, 22, 24], whereas no statistically significant differences have been demonstrated in the response rates between FOLFOXIRI + bevacizumab (63.8%), compared to FOLFIRI + bevacizumab (65.4%), in left-sided *RAS* and *BRAF* wt mCRC [15]. However, propensity score-match analysis showed that bevacizumab + FOLFOXIRI did not increase the response rates [16].

In patients with left-sided *RAS* wt mCRC, when the treatment goal was survival, CT doublet + bevacizumab and CT triplet + anti-EGFR or bevacizumab were not the preferred option in the first-line setting (90%, “consensus”). Toxicity and the current evidence may limit these options [15, 20]. Finally, a meta-analysis has revealed better results in this patient group for the combination of CT + anti-EGFR, compared to CT + bevacizumab, in terms of both OS (HR = 0.75; 95% CI 0.67–0.84) and PFS (HR = 0.78; 95% CI 0.70–0.87) [9].

Full Delphi results are presented in Table 2 of the supplementary material.

### Triplets (Table 3)

With regards to the first-line treatment of patients with *RAS* wt mCRC, the panel considered that, in general, CT triplets + targeted therapy is not more effective than the sequential approach of CT doublets + targeted therapy. No studies have been specifically designed to analyze this question for *RAS* wt patients.

A sub-analysis of the TRIBE-1 study [25] revealed a median OS in *RAS* wt mCRC patients of 26.8 months with FOLFIRI + bevacizumab, compared to 37.1 months with FOLFOXIRI + bevacizumab (HR = 0.78; *p* = 0.66).

The TRIBE-2 study was designed to compare whether three CT + bevacizumab, followed by maintenance strategy with 5-FU/bevacizumab and their reintroduction after the progression (Arm B), was more effective than a pre-planned use of the same agents in consecutive therapy lines (Arm A) [26]. The primary outcome was PFS2, defined as the time from randomization to progression on any treatment given after first progression or death. Using triplets/bevacizumab was statistically significantly associated with PFS2 improvement (19.1 vs. 16.4 months, *p* < 0.001), response rate (62% vs. 50%, *p* = 0.002), first PFS (12.0 vs. 9.8 months, *p* < 0.001), and OS (27.6 vs. 22.6 months, *p* = 0.033). However, no specific sub-analysis for the *RAS* wt population is available.

Similarly, most experts agreed that the first-line use of triplet CT + targeted therapy is conditioned by hard-to-handle toxicity (73%, “majority”) since this would limit the therapeutic options of second-line treatment (70%, “majority”).

In clinical trials, using triplet CT is associated with a significant increase in Grade 3–4 toxicity. FOLFOXIRI + bevacizumab has been associated with a significantly higher rate of serious adverse events, including neutropenia (50%), febrile neutropenia (8.8–7.2%), and diarrhea (17%) [26, 27]. Reported rates with FOLFOXIRI + cetuximab were 31% for neutropenia, 3% for fever, 18% for diarrhea, and 16% for skin toxicity [24]. In the VOLFI trial’s [28], under FOLFOXIRI + panitumumab, Grade 3–4 events were observed in 80.3% of patients.

Many experts agreed that using triplet CT + biologic therapies in first-line treatment might limit the use of active therapies in subsequent treatment lines. A limited number of first-line cycles, as in the TRIBE and TRIBE-2 trials [25, 26], along with the use of maintenance therapy, facilitates the availability of effective second-line treatments. In the TRIBE study, 76% of patients in both treatment arms received second-line treatment at disease progression (*p* = 0.92) [27]. In the TRIBE-2 trial, 86% of patients treated in Arm A received second-line treatment, of whom 78% were treated with FOLFIRI + bevacizumab, according to the study design. In Arm B, 81% of patients received second-line treatment at first disease progression [26].

There was a broad consensus among experts about the appropriate use of anti-EGFR drugs in the second-line setting (93%, “consensus”), following triplet CT triplet + bevacizumab in *RAS* wt. Experts also broadly agreed on using antiangiogenic drugs following progression to first-line treatment with triplet CT + anti-EGFR therapy (97%, “consensus”).

However, efficacy data have not been published for either strategy. In the TRIBE trial [27], 29% of patients on first-line triplet CT triplet + bevacizumab received an anti-EGFR agent in the second-line setting, and 29% of patients continued with bevacizumab at disease progression. In 13% of patients, anti-EGFR therapy was administered in the third-line setting. In the TRIBE-2 trial [26], 59% of patients in Arm B received the pre-planned reintroduction of FOLFOXIRI + bevacizumab in the second-line setting. Overall, 9% received triplet CT without biologic therapy, and only 4% received CT with anti-EGFR drugs. Both studies included patients irrespective of their *RAS* mutational status. Moreover, the rate of *RAS* wt patients on anti-EGFR therapy was not reported.

Most experts (93%) asserted that treatment goal influences the use of CT triplets + targeted therapy in the first line for patients with *RAS* wt mCRC, particularly when the goal is response or tumor resectability [15, 27, 28].

A pooled analysis of mCRC patients with unresectable liver-limited metastases was subsequently performed. A total of 42% of patients were *RAS* wt that had been treated with first-line FOLFOXIRI + bevacizumab [29]. The objective response rate (ORR) was 69%, and a R0/R1 surgical resection of metastases was possible in 36.1% of patients. The mPFS in resected patients was 18.1 months and the mOS 44.3 months. The Phase II OLIVIA trial [30] randomized patients with unresectable liver metastases into FOLFOXIRI + bevacizumab or mFOLFOX-6 + bevacizumab, in the context of conversion to surgery. The *RAS* mutational status was not evaluated. The authors reported a response rate of 81%, and 49% of R0 resection rates in the CT triplet + bevacizumab group, compared to 23% of R0 in the other group. In a single-arm Phase II study, the combination of FOLFIRINOX + cetuximab in the first line showed ORR of 70% in unresectable mCRC patients and an R0 conversion rate of 37% in *KRAS* wt patients < 70 years with a PS0-1 [22]. The response rate of FOLFOXIRI + panitumumab versus FOLFOXIRI was the main outcome of the VOLFI trial [28], which included patients with unresectable RAS wt mCRC. This trial also reported significantly higher ORR in the FOLFOXIRI + panitumumab group (87.3% vs. 60.6%; *p* = 0.004). The rate of secondary resection of metastases, as a secondary outcome, was also significantly higher (33% vs. 12.1%; *p* = 0.02) in the full analysis set, which increased to 75% (vs. 36.4%) in patients with a chance of secondary resection with curative intent [31].

As discussed in the previous section, with regards to tumor location, the experts considered the use of a triplet CT with antiangiogenic drugs to be the main treatment option in the first line (80%, “consensus”) for *RAS* wt mCRC patients with a right-sided primary tumor, and potentially resectable disease [15, 27]. Conversely, 57% considered CT triplets + anti-EGFR agents to be an acceptable first option in this subgroup of patients [24, 28].

Likewise, for *RAS* wt mCRC patients with a left-sided primary tumor and potentially resectable disease, in the absence of contraindications, the experts asserted that the combination of a CT triplet with anti-EGFR in the first-line setting was not more appropriate than using a doublet CT + anti-EGFR therapy [17, 28]. This reinforces the messages proposed in the tumor-location section.

Most of the experts (83%, “consensus”) considered triplet CT with antiangiogenic drugs in the first line to be the preferential treatment for *BRAF*-mutated (mt) mCRC patients. The *BRAF* V600 mt is an essential prognosis marker in mCRC patients, as it has been associated with poor response to conventional treatments. The estimated OS rates of *BRAF* V600 mt patients is around 11 months, compared to an average of 35 months in *BRAF* wt patients. Data on the efficacy of anti-EGFR therapy in *RAS* wt/*BRAF* mt mCRC patients were shown worse than those in *RAS* wt/*BRAF* wt patients. These observations encourage a discussion about whether *BRAF* mt is a predictive biomarker of poor response to anti-EGFR therapy [32–35].

The TRIBE-1 trial included 28 *BRAF* V600E mt patients [25]. Compared to *RAS/BRAF* wt patients, *BRAF* mt patients showed significant lower OS (HR = 2; *p* = 0.003). Additionally, those on FOLFOXIRI + bevacizumab (*n* = 16) reported better OS (19 vs. 10.7 months), PFS (7.5 months vs 5.5 months), and response rate (56% vs 46%) than those on FOLFIRI + bevacizumab (*n* = 12) [25, 27]. The results of this sub-analysis positioned FOLFOXIRI + bevacizumab as the clinical guidelines’ first choice for first-line treatment of patients with *BRAF* mt mCRC [8, 14]. The TRIBE-2 trial [26] showed no statistically significant differences in PFS2 between the groups of *BRAF* mt patients. Similarly, in 33 *BRAF* mt patients in the VISNU-1 trial [36], no statistically significant differences in PFS were reported, when comparing FOLFOXIRI + bevacizumab and FOLFOX + bevacizumab.

Conversely, 93% of experts did not consider triplet CT + anti-EGFR therapy to be the preferred option for *BRAF* mt patients, which aligns with the clinical guidelines [8, 14]. There is currently no published data from Phase III RCTs that assess CT triplets with anti-EGFR therapy. The VOLFI trial [28] demonstrated a significant increase for mFOLFOXIRI + panitumumab in the ORR (*p* = 0.04) of 16 *BRAF* mt mCRC patients.

Full Delphi results are presented in Table 3 of the supplementary material.

### Maintenance (Table 4)

Maintenance treatment is defined as the continuation of part of the CT and/or biological treatment that was initially used in the first-line treatment of the mCRC.

There was consensus among experts (97%) that maintenance in the first-line setting for unresectable mCRC patients is standard practice. It is, therefore, recommended in the main clinical guidelines [8].

Several studies have demonstrated the efficacy of maintenance therapy in mCRC patients, mainly using combinations of fluoropyrimidines and bevacizumab [37–40]. The Phase III CAIRO3 trial demonstrated the benefit of maintenance treatment with capecitabine + bevacizumab after six cycles of CAPOX, compared to total treatment suspension, in terms of PFS2 (which was the study’s primary endpoint) [40]. In an update, this benefit continued in the whole population and across all mutational subgroups (*RAS*/*BRAF*/MSI) [39]. Results from Phase II and III trials comparing maintenance therapy with bevacizumab or complete discontinuation of treatment, compared to maintenance with fluoropyrimidines and bevacizumab, showed improved PFS and a trend toward better OS for the latter strategy [37, 38]. This option would also be possible following FOLFOXIRI + bevacizumab.

Several trials have also analyzed maintenance with anti-EGFR agents in *RAS* wt mCRC. The MACRO-2 trial found no significant differences in PFS between maintenance with cetuximab and the continuation of mFOLFOX and cetuximab after induction therapy with eight cycles of mFOLFOX + cetuximab [41]. In the SAPPHIRE trial, after six cycles of mFOLFOX + panitumumab, patients were randomized into mFOLFOX + panitumumab or 5-FU/leucovorin + panitumumab [38], without any differences in PFS and OS observed. In the VALENTINO phase II trial of non-inferiority, maintenance therapy with 5-FU/leucovorin + panitumumab after eight cycles with FOLFOX + panitumumab demonstrated superiority to panitumumab monotherapy in PFS [42].

Conversely, experts did not recommend anti-EGFR (80%) or antiangiogenic (97%) monotherapy as a maintenance treatment in unresectable mCRC.

According to the experts, induction therapy in the first-line treatment for patients with unresectable mCRC should be maintained until the maximum response is achieved (87%, “consensus”) [8]. However, this does not mean that the number of cycles should always be pre-set. Instead, the experts asserted that, in clinical practice, each case must be individualized, and the treatment scheme should be adapted to the patient’s features, treatment goal, response, and toxicity.

Finally, 87% of the experts agreed that the first option in mCRC patients at disease progression after maintenance treatment is the reintroduction of the initial treatment, in the absence of relevant residual toxicity.

Full Delphi results are presented in Table 4 of the supplementary material.

### Second-line treatment and beyond (Table 5)

One of this section’s most essential conclusions is that there is consensus among the experts that the combination of CT + targeted therapy should be part of the second-line treatment of most fit patients with *RA*S wt mCRC, which is also recommended in the main clinical guidelines [8, 14]. The use of targeted therapy at disease progression to the first line was evaluated by meta-analysis and shown associated with improved outcomes [43]. These data, together with those from Phase III trials [44–47], have demonstrated the efficacy of this combination, both for antiangiogenic and anti-EGFR treatment, which supports using second-line targeted therapies.

When selecting a second-line treatment, the experts emphasized that the resectability of the metastatic disease should be a treatment goal throughout the disease course, based on published data that confirm the conversion to resectable disease after second-line treatment [48, 49].

Conversely, 87% of experts did not consider the primary tumor location to be a factor in the selection of second-line treatment. Data from the VELOUR trial [45], which evaluated the efficacy of FOLFIRI + aflibercept in second-line treatment, showed the same benefit in terms of OS in a left-sided primary tumor and right-sided primary tumor. In the Phase III 181 trial [6, 47], the combination of FOLFIRI + panitumumab demonstrated no differences in response rate, SLP, or OS according to the primary tumor location. However, the results were generally worse in the right-sided tumors, which probably reflects the already-acknowledged worse prognosis of right-sided primary tumors.

With regard to treatment sequence, the experts primarily indicated that the optimal sequence is yet to be elucidated, due to a lack of data from RCTs. Ongoing studies (CR-SEQUENCE and STRATEGIC-1) are currently providing new insights into this context. Until they yield more results, treatment selection in the second line is largely determined by the first-line treatment, the patient’s features, and the tumor’s characteristics and molecular profile.

When experts were asked about the preferred sequence for *RAS* wt mCRC patients that had received a first-line anti-EGFR treatment, there was unanimity (100% agreement) about considering an antiangiogenic drug. Three Phase III trials on second-line antiangiogenic treatments are currently being conducted, but none of them include patients with first-line anti-EGFR treatments [44–46]. Retrospective analyses of the FIRE III study support the sequence of anti-EGFR therapy followed by antiangiogenic treatment [4], while other exploratory and pre-clinical data also support this sequence [50–52].

However, for *RAS* wt mCRC patients that have received first-line antiangiogenic treatment, there was no consensus about second-line treatment among the experts. While 60% of experts did not support the sequence of antiangiogenic–antiangiogenic therapy, 56% supported the sequence of antiangiogenic-anti-EGFR therapy as a preferred option. These results align with some available data from Phase III studies in second-line treatment. The VELOUR and TML trials (ML18147) [44, 45] also support the antiangiogenic–antiangiogenic sequence of treatment. The 181 study showed benefits in both response rate and PFS with FOLFIRI + panitumumab in the second-line setting, but not in OS. However, only a small number of patients received first-line antiangiogenic treatment [47]. Data from trials such as FIRE-3 [4], as well as observational and in vitro studies [51, 53], suggest that using first-line antiangiogenic drugs for *RAS* wt mCRC patients would negatively impact the efficacy of anti-EGFR agents in the second-line setting [53]. Moreover, pooled exploratory data from the PRIME, PEAK, and 181 trials depicted a trend (not statistically significant) toward an improved median OS for panitumumab in the first-line treatment, followed by an antiangiogenic therapy in the second-line (36.8 months), compared to first-line bevacizumab and second-line anti-EGFR therapy (27.8 months) [50]. All these controversial data would explain the lack of consensus about the appropriate sequence after antiangiogenic treatment in the first-line.

The use of oncological treatments in the third-line and beyond is a reality for *RAS* wt mCRC patients (100% agreement). Experts considered the cost–benefit balance to be present in the choice of treatment for patients with mCRC beyond the second-line (93% agreement) [54]. There was agreement (80%) that, in *RAS* wt mCRC patients, drug toxicity is the most crucial factor when selecting treatment beyond the second-line. Regorafenib and TAS 102 have demonstrated benefits in terms of OS without impairment to quality of life when compared with best supportive care beyond the second line [55–57].

Furthermore, a broad consensus was reached (97%) concerning *RAS* wt mCRC patients that have received first- and second-line antiangiogenic treatments. In these patients, the preferred option was anti-EGFR therapy with or without CT. Both cetuximab and panitumumab have demonstrated benefits in terms of response rate and PFS without impairment to quality of life, compared with best supportive care [58, 59]. More recently, an updated retrospective analysis from ASPECCT and WJOG6510G has concluded that, even in multivariate analysis, panitumumab was superior to cetuximab in terms of OS for patients that had previously received bevacizumab (HR = 0.69; 95% CI 0.54–0.87) [60].

Finally, the experts also considered (93% agreement) it important to have MSI and *HER2* data in clinical practice, for patients with *RAS* wt mCRC, to aid treatment decision making in the third-line and beyond. Molecular studies suggest that amplification of the *HER2* gene may be involved in resistance to anti-EGFR antibodies in patients with *RAS* or *BRAF* wt mCRC [61, 62]. Other studies have shown the benefits of immunotherapy in patients with high MSI. Although these drugs are not approved in mCRC, analysis of the molecular profile is necessary because these patients might benefit from their inclusion in clinical trials.

Full Delphi results are shown in Table 5 of the supplementary material.

### Retreatment and liquid biopsy (Table 6)

Rechallenge with anti-EGFR therapies was defined as the re-use of these agents after a period of time without them (ideally more than 6 months), provided that the patient has shown previous benefits and has progressed on treatment with anti-EGFR antibodies.

Reintroduction with anti-EGFR therapies was defined as the re-use of these treatments in patients that have not developed resistance to them or have not progressed during their use.

The experts noted that, for patients with *RAS* wt mCRC, assessment of *RAS* mutational status (exons 2, 3, and 4 of *KRAS* and *NRAS*) by liquid biopsy is a standard alternative that is comparable to paraffin (80% of agreement). Moreover, 100% considered it a standard alternative in the case of insufficient availability of paraffin material. Several studies have been published that have analyzed the validity of liquid biopsy and its possible utility when a rechallenge with anti-EGFR antibodies is considered [63–65].

There was unanimity (100% agreement) that the sensitivity of the technique used for the liquid biopsy is a critical decision-making factor in *RAS* wt mCRC. Several similar studies have compared the ability of liquid biopsy to establish the *RAS* mutational state, compared to solid biopsy. Depending on the technique used, the sensitivity of the liquid biopsy varied from 85% to 94%, with a specificity of approximately 90% and high concordance (even higher than 90%) [63, 64].

The panel also agreed (87%) that, in patients with *RAS* wt mCRC, liquid biopsy determinations should be performed exclusively in experienced reference centers (87%).

Conversely, 67% of experts did not consider rechallenge with anti-EGFR therapies a comparable alternative to approved standard therapies in patients that have received all therapies considered standard in *RAS* wt disease. The Phase II CRICKET trial [66] evaluated 28 *RAS* and *BRAF* wt mCRC patients that were initially sensitive to and later resistant to first-line irinotecan- and cetuximab-based therapy, followed by second-line oxaliplatin- and bevacizumab-based treatment. The efficacy of the rechallenge of a regimen that included cetuximab and irinotecan was subsequently tested. The overall response was 21%, and 46% of patients progressed. The PFS was significantly higher in the *RAS* wt subgroup of patients, compared to the mutated patients, but there were no significant differences in terms of OS between the groups. Pooled data from the PEAK and PRIME studies of *RAS* wt mCRC patients that had undergone EGFR-inhibitor rechallenge had a median OS after rechallenge of 14.2 months and a median OS from the start of treatment of more than 45 months [67]. According to the experts, this evidence is only preliminary. However, if rechallenge with anti-EGFR therapies is considered for patients that have received all therapies that are considered standard, 90% of the experts asserted that liquid biopsy should be used to make the final decision.

With regards to anti-EGFRs reintroduction, 70% of experts considered this a comparable alternative to approved standard therapies for patients that have received all therapies that are considered standard in *RAS* wt disease. Furthermore, 90% noted that liquid biopsy should be used for decision making when considering anti-EGFR reintroduction.

Finally, 97% of the experts agreed on the importance of defining the exact cut-off for clinically relevant *RAS*-mutant allele frequencies in liquid biopsy; that is to say, the mutant allele fraction (MAF) below which a patient is understood to still benefit from anti-EGFR treatment. Although robust and definitive evidence is lacking, the panel recognized the clinical significance of the cut-off points. PERSEIDA was an observational prospective study in patients with *RAS* wt mCRC, most of whom were on panitumumab. In this study, liquid biopsies were collected at 20 ± 2 weeks and at disease progression [65, 68]. MAF cut-offs of ≥ 1%, ≥ 0.1%, and ≥ 0.02% were considered, whose negative correlations with tumor tissue biopsy were 97.5%, 95%, and 87.4%, respectively.

Full Delphi results are presented in Table 6 of the supplementary material.

## Discussion

This consensus reinforces and complements recommendations provided by ESMO and SEOM guidelines regarding the management of *RAS* wt mCRC patients [8, 14]. We identified the most relevant questions and controversies in this field, critically evaluated the available evidence, and provided oncologists with specific information in the form of several statements that had undergone a Delphi process.

We would like to highlight several points. First, tumor sidedness should be taken into account when selecting a first-line treatment. Evidence indicates that first-line CT + anti-EGFR therapy is a preferred option in left-sided primary tumors of *RAS* wt mCRC patients [17–22, 24], but the effect of this treatment strategy in right-sided primary tumors remains unclear [9].

Subsequently, the use of triplet CT is definitively influenced by treatment goal (particularly when respectability is considered), as well as toxicity and the impact of this strategy on second-line treatment options. Similarly, tumor location led to a specific treatment proposal in the first line when using triplet CT, as previously explained. However, we would like to note that, although the available evidence from triplet CT + anti-EGFR therapy in the first-line is promising, it is still only initial [22, 28].

With regards to maintenance, containing targeted therapies in the first-line treatment of unresectable mCRC patients is considered standard in clinical practice, which is also supported by evidence [37–41]. However, conversely, a complete cessation of first-line CT, biologicals, and intermittent schemes are not preferred alternatives to maintenance.

It should also be noted that, in the second line, anti-EGFR drugs can be considered after progression to a first-line triplet CT triplet + antiangiogenic therapy, as well as antiangiogenic drugs after progression to a triplet + antiEGFR. Nevertheless, the best treatment strategy and sequence are currently unknown. In the second-line and beyond, the experts would like to encourage oncologists to include many (but not all) factors in their decision making that are considered in the first-line. This includes response as a treatment goal, particularly in fit patients. This is also true of CT plus targeted therapy, as robust evidence supports their efficacy in this context [43–47, 69]. Additionally, new molecular evidence [61, 62, 70] encourages including assessment of MSI and *HER2* mutational status in treatment decision making for the third-line and beyond.

Finally, although liquid biopsy is an emerging technique, the experts declared that the assessment of *RAS* mutational status in mCRC using liquid biopsy is a standard alternative that is comparable to paraffin. Moreover, although more research is needed, a liquid biopsy should be performed, when anti-EGFR therapy rechallenge is considered [63–65].

In summary, although new and robust evidence is needed to definitively clarify some of the issues that are developed in the present document, we believe that the practical framework provided in this document will help oncologists manage *RAS* wt mCRC patients.

## Electronic supplementary material

Below is the link to the electronic supplementary material.

Supplementary material 1 (DOC 222 kb)
