# Supplementary material for: Controversies in the treatment of RAS wild-type metastatic colorectal cancer
Source: Clin Transl Oncol. 2020 Aug 13;23(4):827–39. doi: 10.1007/s12094-020-02475-8 (PMC7979622; doi:10.1007/s12094-020-02475-8)
Supplement: Supplementary file 1 — Supplementary material 1 (DOC 222 kb) [file 12094_2020_2475_MOESM1_ESM.doc]

Tables a to f depict the results of the two Delphi processes of the whole project according to the six different sections.

**Table a**. Overarching principles.

| # | Statement | Delphi round | Range of response (n=30)* | | | | % of panel^†^ | Final consensus^‡^ |
| --- | --- | --- | --- | --- | --- | --- | --- | --- |
|  |  |  | 1 (%) | 2 (%) | 3 (%) | 4 (%) |  |  |
| 1 | In *RAS* wt mCRC patients, *RAS/BRAF* mutational status and the microsatellite instability (MSI) assessment are strongly recommended to appropriately select a treatment | 1 | 97 | 3 | 0 | 0 | 100 | Unanimous (agreement) |
|  |  | 2 | - | - | - | - | - | - |
| 2 | In *RAS* wt mCRC patients, the best first-line treatment option is the combination of CT and biologic therapy, in fit patients. | 1 | 90 | 10 | 0 | 0 | 100 | Unanimous (agreement) |
|  |  | 2 | - | - | - | - | - | - |
| 3 | In *RAS* wt mCRC patients, care should be provided by a multidisciplinary team | 1 | 93 | 7 | 0 | 0 | 100 | Unanimous (agreement) |
|  |  | 2 | - | - | - | - | - | - |

**Abbreviations:** wt=wild-type; mCRC=metastatic colorectal cancer; CT=chemotherapy.

*Delphi 1^st^ and 2^nd^ round response categories: 1=totally agree; 2=basically agree; 3=basically disagree; 4=totally disagree.

†Rate of respondents who voted agree (1 or 2) or disagree (3 or 4) after each Delphi round.

‡Final consensus was defined as: “unanimous” when the response rate in categories 1 and 2 or 3 or 4 was 100%; “consensus” when the response rate in categories 1 and 2 or 3 and 4 was 75-99%; “majority” when the response rate in categories 1 and 2 or 3 and 4 was 60%-74%; “dissent” if the response rate was <60%.

**Table b. Primary tumor sidedness.**

| # | Statement | Delphi round | Range of response (n=30)* | | | | % of panel^†^ | Final consensus^‡^ |
| --- | --- | --- | --- | --- | --- | --- | --- | --- |
|  |  |  | 1 (%) | 2 (%) | 3 (%) | 4 (%) |  |  |
| 4 | In *RAS* wt mCRC patients, primary tumor location influences first-line treatment selection in mCRC | 1 | 27 | 60 | 10 | 3 | 87 | Consensus (agreement) |
|  |  | 2 | 7 | 83 | 7 | 3 | 90 |  |
| 5 | In *RAS* wt mCRC patients, the primary tumor location is more relevant than the tumor molecular profile when selecting the first-line treatment in mCRC | 1 | 3 | 13 | 37 | 47 | 84 | Consensus (disagreement) |
|  |  | 2 | 0 | 3 | 47 | 50 | 97 |  |
| 6 | In *RAS* wt mCRC patients and right-sided primary tumor, the first-line treatment selection should take into account the treatment goal (response vs. survival) | 1 | 37 | 40 | 20 | 3 | 77 | Consensus (agreement) |
|  |  | 2 | 43 | 54 | 0 | 3 | 97 |  |
|  | In *RAS* wt mCRC patients with right-sided primary tumor and response as treatment goal, the preferred first-line treatment, in the absence of contraindications, would be: |  | | | | | | |
| 7 | CT doublet + anti-EGFR | 1 | 27 | 33 | 37 | 3 | 60 | Majority  (agreement) |
|  |  | 2 | 20 | 40 | 37 | 3 | 60 |  |
| 8 | CT triplet + anti-EGFR | 1 | 13 | 13 | 44 | 30 | 74 | Consensus (disagreement) |
|  |  | 2 | 7 | 17 | 63 | 13 | 76 |  |
| 9 | CT doublet + bevacizumab | 1 | 7 | 33 | 37 | 23 | 60 | Majority  (disagreement) |
|  |  | 2 | 7 | 30 | 50 | 13 | 63 |  |
| 10 | CT triplet + bevacizumab | 1 | 43 | 37 | 13 | 7 | 80 | Consensus (agreement) |
|  |  | 2 | 50 | 30 | 20 | 0 | 80 |  |
|  | In *RAS* wt mCRC patients with right-sided primary tumor and survival as treatment goal, the preferred first-line treatment, in the absence of contraindications, would be: |  | | | | | | |
| 11 | CT doublet + anti-EGFR | 1 | 3 | 23 | 34 | 40 | 74 | Consensus (disagreement) |
|  |  | 2 | 7 | 6 | 27 | 60 | 87 |  |
| 12 | CT triplet + anti-EGFR | 1 | 3 | 4 | 10 | 83 | 93 | Consensus (disagreement) |
|  |  | 2 | - | - | - | - | - |  |
| 13 | CT doublet + bevacizumab | 1 | 57 | 33 | 7 | 3 | 90 | Consensus (agreement) |
|  |  | 2 | 60 | 33 | 7 | 0 | 93 |  |
| 14 | CT triplet + bevacizumab | 1 | 13 | 50 | 17 | 20 | 63 | Majority  (agreement) |
|  |  | 2 | 7 | 63 | 23 | 7 | 70 |  |
| 15 | In *RAS* wt mCRC patients with left-sided primary tumor, the selection first-line treatment should take into account the treatment goal (response vs. survival) | 1 | 37 | 17 | 33 | 13 | 54 | Majority  (agreement) |
|  |  | 2 | 57 | 3 | 37 | 3 | 60 |  |
|  | In *RAS* wt mCRC patients with left-sided primary tumor and response as treatment goal, the preferred first-line treatment, in absence of contraindications, would be: |  |  |  |  |  |  |  |
| 16 | CT doublet + anti-EGFR | 1 | 93 | 0 | 7 | 0 | 93 | Consensus (agreement) |
|  |  | 2 | - | - | - | - | - |  |
| 17 | CT triplet + anti-EGFR | 1 | 17 | 33 | 27 | 23 | 50 | Dissent |
|  |  | 2 | 10 | 37 | 40 | 13 | 53 |  |
| 18 | CT doublet + Bevacizumab | 1 | 7 | 17 | 30 | 46 | 76 | Consensus (disagreement) |
|  |  | 2 | 3 | 7 | 27 | 63 | 90 |  |
| 19 | CT triplet + Bevacizumab | 1 | 13 | 33 | 37 | 17 | 54 | Dissent |
|  |  | 2 | 7 | 37 | 33 | 23 | 56 |  |
|  | In *RAS* wt mCRC patients with left-sided primary tumor and survival as treatment goal, the preferred first-line treatment, in the absence of contraindications, would be: |  | | | | | | |
| 20 | CT doublet + anti-EGFR | 1 | 83 | 10 | 7 | 0 | 93 | Consensus (agreement) |
|  |  | 2 | - | - | - | - | - |  |
| 21 | CT triplet + anti-EGFR | 1 | 3 | 27 | 17 | 57 | 74 | Consensus (disagreement) |
|  |  | 2 | 0 | 13 | 10 | 77 | 87 |  |
| 22 | CT doublet + bevacizumab | 1 | 14 | 23 | 23 | 40 | 63 | Consensus (disagreement) |
|  |  | 2 | 7 | 10 | 33 | 50 | 83 |  |
| 23 | CT triplet +b | 1 | 3 | 17 | 27 | 53 | 80 | Consensus (disagreement) |
|  |  | 2 | 3 | 10 | 27 | 60 | 87 |  |

**Abbreviations:** wt=wild-type; mCRC=metastatic colorectal cancer; CT=chemotherapy; EGFR= epidermal growth factor receptor.

*Delphi 1^st^ and 2^nd^ round response categories: 1=totally agree; 2=basically agree; 3=basically disagree; 4=totally disagree.

†Rate of respondents who voted agree (1 or 2) or disagree (3 or 4) after each Delphi round.

‡Final consensus was defined as: “unanimous” when the response rate in categories 1 and 2 or 3 or 4 was 100%; “consensus” when the response rate in categories 1 and 2 or 3 and 4 was 75-99%; “majority” when the response rate in categories 1 and 2 or 3 and 4 was 60%-74%; “dissent” if the response rate was <60%.

**Table c. Triplets.**

| # | Statement | Delphi round | Range of response (n=30)* | | | | % of panel^†^ | Final consensus^‡^ |
| --- | --- | --- | --- | --- | --- | --- | --- | --- |
|  |  |  | 1 (%) | 2 (%) | 3 (%) | 4 (%) |  |  |
| 24 | In general, in *RAS* wt mCRC patients, first-line treatment with CT triplets + targeted therapy is more effective than the sequential use of CT doubles + targeted therapy | 1 | 7 | 30 | 36 | 27 | 63 | Majority (disagreement) |
|  |  | 2 | 0 | 27 | 46 | 27 | 73 |  |
| 25 | In *RAS* wt mCRC patients, CT triplets + targeted therapy in first-line is conditioned for it limits treatment options in the second-line setting | 1 | 13 | 50 | 20 | 17 | 63 | Majority  (agreement) |
|  |  | 2 | 3 | 67 | 17 | 13 | 70 |  |
| 26 | In *RAS* wt mCRC patients, CT triplets + targeted therapy in first-line is conditioned by hard-to-handle toxicity | 1 | 17 | 46 | 27 | 10 | 63 | Majority  (agreement) |
|  |  | 2 | 3 | 70 | 27 | 0 | 73 |  |
| 27 | In *RAS* wt mCRC patients, the treatment goal determines the use of first-line CT triplets + targeted therapy | 1 | 40 | 30 | 27 | 3 | 70 | Consensus (agreement) |
|  |  | 2 | 67 | 30 | 3 | 0 | 97 |  |
| 28 | In *RAS* wt mCRC patients, a first-line CT triplet + targeted therapy should be considered as first option in patients with unresectable metastatic disease | 1 | 13 | 17 | 30 | 40 | 70 | Consensus (disagreement) |
|  |  | 2 | 13 | 7 | 33 | 47 | 77 |  |
| 29 | In *RAS* wt mCRC patients with a right-sided primary tumor potentially resectable disease, a first-line CT triplet + antiangiogenic therapy should be considered as aa first option | 1 | 43 | 30 | 17 | 10 | 73 | Consensus (agreement) |
|  |  | 2 | 57 | 23 | 20 | 0 | 80 |  |
| 30 | In *RAS* wt mCRC patients with a right-sided primary tumor and potentially resectable disease, a first-line CT triplet + anti-EGFR antibodies should be considered as a first option the use of | 1 | 27 | 23 | 23 | 27 | 50 | Dissent |
|  |  | 2 | 34 | 23 | 23 | 20 | 57 |  |
| 31 | In *RAS* wt mCRC patients with a left-sided primary tumor and potentially resectable disease, in the absence of contraindications, it is more appropriate to associate a CT triplet with anti-EGFR in the first line than the use of CT doublet + anti-EGFR | 1 | 10 | 20 | 47 | 23 | 60 | Consensus (disagreement) |
|  |  | 2 | 10 | 13 | 64 | 13 | 77 |  |
| 32 | In *RAS* wt mCRC patients, first-line CT triplet + antiangiogenic therapy is preferred in *BRAF* mt patients | 1 | 43 | 44 | 10 | 3 | 77 | Consensus (agreement) |
|  |  | 2 | 40 | 43 | 17 | 0 | 83 |  |
| 33 | In *RAS* wt mCRC patients, first-line CT triplet + anti-EGFR therapy is preferred in *BRAF* mt patients | 1 | 3 | 4 | 23 | 70 | 93 | Consensus (disagreement) |
|  |  | 2 | 0 | 6 | 17 | 77 | 94 |  |
| 34 | In *RAS* wt mCRC patients after using a first-line CT triplet + antiangiogenic therapy, anti-EGFR drugs are considered appropriate for the second-line treatment | 1 | 67 | 20 | 10 | 3 | 87 | Consensus (agreement) |
|  |  | 2 | 77 | 17 | 3 | 3 | 94 |  |
| 35 | In *RAS* wt mCRC patients after progression to a first-line CT triplet + anti-EGFR therapy, the use antiangiogenic drugs in second line of treatment is considered appropriate | 1 | 60 | 30 | 10 | 0 | 90 | Consensus (agreement) |
|  |  | 2 | 83 | 14 | 3 | 0 | 97 |  |

**Abbreviations:** wt=wild-type; mCRC=metastatic colorectal cancer; CT=chemotherapy; EGFR= epidermal growth factor receptor; mt=mutated.

*Delphi 1^st^ and 2^nd^ rounds response categories: 1=totally agree; 2=basically agree; 3=basically disagree; 4=totally disagree.

†Rate of respondents who voted agree (1 or 2) or disagree (3 or 4) after each Delphi round.

‡Final consensus was defined as: “unanimous” when the response rates in categories 1 and 2 or 3 or 4 was 100%; “consensus” when the response rates in categories 1 and 2 or 3 and 4 was 75-99%; “majority” when the response rate in categories 1 and 2 or 3 and 4 was 60%-74%; “dissent” if the response rate was <60%.

**Table d. Maintenance treatment.**

| # | Statement | Delphi round | Range of response (n=30)* | | | | % of panel^†^ | Final consensus^‡^ |
| --- | --- | --- | --- | --- | --- | --- | --- | --- |
|  |  |  | 1 (%) | 2 (%) | 3 (%) | 4 (%) |  |  |
| 36 | In *RAS* wt mCRC patients with unresectable disease, maintenance treatment in the first-line setting is a standard practice | 1 | 67 | 17 | 16 | 0 | 84 | Consensus (agreement) |
|  |  | 2 | 93 | 3 | 4 | 0 | 96 |  |
| 37 | In *RAS* wt mCRC patients with unresectable disease, complete treatment cessation (CT and biologicals) after reaching maximum response is a preferred alternative to maintenance treatment in the first-line setting | 1 | 3 | 7 | 40 | 50 | 90 | Consensus (disagreement) |
|  |  | 2 | 3 | 3 | 30 | 64 | 94 |  |
| 38 | In *RAS* wt mCRC patients with unresectable desease, intermittent treatment with pre-established periods of rest for both, CT and biological therapy, is a preferred alternative to maintenance treatment in the first-line setting | 1 | 3 | 10 | 43 | 44 | 87 | Consensus (disagreement) |
|  |  | 2 | 3 | 3 | 44 | 50 | 94 |  |
| 39 | In *RAS* wt mCRC patients with unresectable disease, first-line induction therapy should be maintained until the maximum response is achieved, in the absence of toxicity that contraindicates it | 1 | 53 | 30 | 10 | 7 | 83 | Consensus (agreement) |
|  |  | 2 | 70 | 17 | 10 | 3 | 87 |  |
| 40 | In *RAS* wt mCRC patients with unresectable disease, when first-line treatment is initiated, it would be desirable to have a pre-established number of induction cycles to be administered | 1 | 13 | 37 | 40 | 10 | 50 | Dissent |
|  |  | 2 | 7 | 37 | 43 | 13 | 56 |  |
| 41 | In *RAS* wt mCRC patients with unresectable disease, when first-line treatment with CT + bevacizumab is initiated, it would be desirable to maintain treatment with fluoropyrimidines + bevacizumab until disease progression | 1 | 67 | 20 | 13 | 0 | 87 | Consensus (agreement) |
|  |  | 2 | 83 | 14 | 3 | 0 | 97 |  |
| 42 | In *RAS* wt mCRC patients, in the first-line treatment using CT and EGFR inhibitors, in the absence of unmanageable toxicity, EGFR inhibitors should be maintained until disease progression | 1 | 44 | 23 | 20 | 13 | 67 | Consensus (agreement) |
|  |  | 2 | 63 | 20 | 7 | 10 | 83 |  |
| 43 | In *RAS* wt mCRC patients with unresectable disease, a first-line maintenance therapy with anti-EGFR in monotherapy is considered a valid treatment option | 1 | 3 | 30 | 44 | 23 | 67 | Consensus (disagreement) |
|  |  | 2 | 0 | 20 | 53 | 27 | 80 |  |
| 44 | In *RAS* wt mCRC patients with unresectable disease, a first-line maintenance therapy with anti-VEGF in monotherapy is consideres a valid treatment option | 1 | 0 | 3 | 20 | 77 | 97 | Consensus (disagreement) |
|  |  | 2 | - | - | - | - | - |  |
| 45 | In *RAS* wt mCRC patients, the first option at disease progression after maintenance treatment is the reintroduction of the initial induction treatment, in the absence of relevant residual toxicity | 1 | 23 | 50 | 23 | 4 | 73 | Consensus (agreement) |
|  |  | 2 | 17 | 70 | 13 | 0 | 87 |  |
| 46 | In *RAS* wt mCRC patients with unresectable disease on FOLFOXIRI + bevacizumab, and after the induction period, maintenance should be performed with fluoropyrimidine ± bevacizumab | 1 | 63 | 27 | 7 | 3 | 90 | Unanimous (agreement) |
|  |  | 2 | 83 | 17 | 0 | 0 | 100 |  |

**Abbreviations:** wt=wild-type; mCRC=metastatic colorectal cancer; CT=chemotherapy; EGFR= epidermal growth factor receptor; VEGF=vascular endothelial growth factor.

*Delphi 1^st^ and 2^nd^ rounds response categories: 1=totally agree; 2=basically agree; 3=basically disagree; 4=totally disagree.

†Rate of respondents who voted agree (1 or 2) or disagree (3 or 4) after each Delphi round.

‡Final consensus was defined as: “unanimous” when the response rate in categories 1 and 2 or 3 or 4 was 100%; “consensus” when the response rate in categories 1 and 2 or 3 and 4 was 75-99%; “majority” when the response rate in categories 1 and 2 or 3 and 4 was 60%-74%; “dissent” if the response rate was <60%.

**Table e. Second-line treatment and beyond.**

| # | Statement | Delphi round | Range of response (n=30)* | | | | % of panel^†^ | Final consensus^‡^ |
| --- | --- | --- | --- | --- | --- | --- | --- | --- |
|  |  |  | 1 (%) | 2 (%) | 3 (%) | 4 (%) |  |  |
| 47 | In *RAS* wt mCRC patients, the combination of CT + targeted therapy should be part of second-line treatment in most fit patients | 1 | 80 | 17 | 10 | 3 | 97 | Consensus (agreement) |
|  |  | 2 | - | - | - | - | - |  |
| 48 | In *RAS* wt mCRC patients, the response rate for a potential resectability is a goal to be considered when selecting second-line treatments | 1 | 47 | 37 | 13 | 3 | 84 | Consensus (agreement) |
|  |  | 2 | 50 | 37 | 10 | 3 | 87 |  |
| 49 | In *RAS* wt mCRC patients, the response rate in terms of symptom control is an objective to be considered when selecting second-line treatments | 1 | 37 | 43 | 13 | 7 | 80 | Consensus (agreement) |
|  |  | 2 | 23 | 73 | 4 | 0 | 96 |  |
| 50 | In *RAS* wt mCRC patients, primary tumor location influences treatment selection in second-line treatment | 1 | 10 | 17 | 30 | 43 | 73 | Consensus (disagreement) |
|  |  | 2 | 3 | 10 | 30 | 57 | 87 |  |
| 51 | In *RAS* wt mCRC patients who have received first-line antiangiogenic treatment, second-line treatment consists of antiangiogenic therapy (1^st^ anti-VEGF, 2^nd^ anti-VEGF) | 1 | 17 | 27 | 43 | 13 | 56 | Majority (disagreement) |
|  |  | 2 | 17 | 23 | 43 | 17 | 60 |  |
| 52 | In *RAS* wt mCRC patients who have received first- and second-line antiangiogenic treatments, the treatment for subsequent lines consists of anti-EGFR (1^st^ anti-VEGF, 2^nd^ anti-VEGF, 3^rd^ anti-EGFR) | 1 | 80 | 17 | 3 | 0 | 97 | Consensus (agreement) |
|  |  | 2 | - | - | - | - | - |  |
| 53 | In *RAS* wt mCRC patients who have received first-line antiangiogenic therapy, anti-EGFR therapy is the preferred treatment in second-line (1^st^ anti-VEGF, 2^nd^ anti-EGFR) | 1 | 33 | 23 | 27 | 17 | 56 | Dissent |
|  |  | 2 | 33 | 23 | 27 | 17 | 56 |  |
| 54 | In *RAS* wt mCRC patients who have been treated with anti-EGFR therapy in first-line, the preferred treatment in second-line is antiangiogenic therapy (1^st^ anti-EGFR, 2^nd^ anti-VEGF) | 1 | 90 | 10 | 0 | 0 | 100 | Unanimous (agreement) |
|  |  | 2 | - | - | - | - | - |  |
| 55 | In *RAS* wt mCRC patients who have not received anti-EGFR treatment in either first- or second-linetreatment, the preferred option in the third-line and beyond is anti-EGFR ± CT | 1 | 83 | 14 | 3 | 0 | 97 | Consensus (agreement) |
|  |  | 2 | - | - | - | - | - |  |
| 56 | In *RAS* wt mCRC patients, using oncological treatment in third-line and beyond is a reality | 1 | 90 | 10 | 0 | 0 | 100 | Unanimous (agreement) |
|  |  | 2 | - | - | - | - | - |  |
| 57 | The cost-benefit balance must be considered when selecting treatments of beyond second-linetreatment | 1 | 60 | 33 | 7 | 0 | 93 | Consensus (agreement) |
|  |  | 2 | 67 | 27 | 6 | 0 | 94 |  |
| 58 | In *RAS* wt mCRC patients, toxicity is the main factor in selecting treatments beyond second-line | 1 | 13 | 57 | 27 | 3 | 70 | Consensus (agreement) |
|  |  | 2 | 3 | 77 | 13 | 7 | 80 |  |
| 59 | In clinical practice, it would be relevant to have MSI and *HER2* data available for the decision-making in third-line treatments and beyond | 1 | 77 | 17 | 6 | 0 | 94 | Consensus (agreement) |
|  |  | 2 | - | - | - | - | - |  |

**Abbreviations:** wt=wild-type; mCRC=metastatic colorectal cancer; CT=chemotherapy; EGFR= epidermal growth factor receptor; VEGF=vascular endothelial growth factor; MSI=microsatellite instability.

*Delphi 1^st^ and 2^nd^ rounds response categories: 1=totally agree; 2=basically agree; 3=basically disagree; 4=totally disagree.

†Rate of respondents who voted agree (1 or 2) or disagree (3 or 4) after each Delphi round.

‡Final consensus was defined as: “unanimous” when the response rate in categories 1 and 2 or 3 or 4 was 100%; “consensus” when the response rate in categories 1 and 2 or 3 and 4 was 75-99%; “majority” when the response rate in categories 1 and 2 or 3 and 4 was 60%-74%; “dissent” if the response rate was <60%.

**Table f. Retreatment and liquid biopsy.**

| # | Statement | Delphi round | Range of response (n=30)* | | | | % of panel^†^ | Final consensus^‡^ |
| --- | --- | --- | --- | --- | --- | --- | --- | --- |
|  |  |  | 1 (%) | 2 (%) | 3 (%) | 4 (%) |  |  |
| 60 | In *RAS* wt mCRC patients, the assessment of *RAS* mutational status (exons 2, 3, 4 of *KRAS* and *NRAS*) by liquid biopsy is a standard alternative to paraffin | 1 | 17 | 50 | 23 | 10 | 67 | Consensus (agreement) |
|  |  | 2 | 10 | 70 | 10 | 10 | 80 |  |
| 61 | In *RAS* wt mCRC patients, the assessment of *RAS* mutational status (exons 2, 3, 4 of *KRAS* and *NRAS*) by liquid biopsy is a standard alternative when sufficient paraffin material is not available | 1 | 67 | 33 | 0 | 0 | 100 | Unanimous (agreement) |
|  |  | 2 | 90 | 10 | 0 | 0 | 100 |  |
| 62 | In *RAS* wt mCRC patients, the assessment of *RAS* mutational status (exons 2, 3, 4 of *KRAS* and *NRAS*) should be performed using only one technique (liquid biopsy or paraffin) | 1 | 10 | 20 | 30 | 40 | 70 | Consensus (disagreement) |
|  |  | 2 | 0 | 3 | 27 | 70 | 97 |  |
| 63 | In *RAS* wt mCRC patients, the detection sensitivity of the liquid biopsy technique is a decisive factor in the decision-making | 1 | 47 | 47 | 3 | 3 | 94 | Unanimous (agreement) |
|  |  | 2 | 53 | 47 | 0 | 0 | 100 |  |
| 64 | In *RAS* wt mCRC patients, liquid biopsy determinations should be performed exclusively at experienced reference centers | 1 | 47 | 36 | 10 | 7 | 83 | Consensus (agreement) |
|  |  | 2 | 63 | 23 | 7 | 7 | 84 |  |
| 65 | Rechallenge with anti-EGFR therapies is a comparable alternative to approved standard therapies in patients who have received all therapies considered standard in *RAS* wt disease | 1 | - | - | - | - | - | Majority  (disagreement) |
|  |  | 2 | 13 | 27 | 47 | 13 | 60 |  |
| 66 | Currently, there is enough scientific evidence to support the rechallenge with anti-EGFR therapies in patients who have received all therapies considered standard in *RAS* wt disease | 1 | - | - | - | - | - | Majority  (disagreement) |
|  |  | 2 | 3 | 23 | 64 | 10 | 74 |  |
| 67 | Liquid biopsy should be used in the decision-making when considering rechallenge with anti-EGFR therapies in patients who have received all therapies considered standard | 1 | - | - | - | - | - | Consensus (agreement) |
|  |  | 2 | 77 | 13 | 3 | 7 | 90 |  |
| 68 | Reintroduction of anti-EFGR therapies is a comparable alternative to approved standard therapies in patients who have received all therapies considered standard in *RAS* wt disease | 1 | - | - | - | - | - | Majority  (agreement) |
|  |  | 2 | 20 | 50 | 27 | 3 | 70 |  |
| 69 | Currently, there is enough scientific evidence to support the reintroduction of anti-EGFR therapies in patients who have received all therapies considered standard in *RAS* wt disease | 1 | - | - | - | - | - | Dissent |
|  |  | 2 | 7 | 40 | 46 | 7 | 53 |  |
| 70 | Liquid biopsy should be used in the decision-making when considering the reintroduction of anti-EGFR therapies in patients who have received all therapies considered standard | 1 | - | - | - | - | - | Consensus (agreement) |
|  |  | 2 | 67 | 23 | 7 | 3 | 90 |  |
| 71 | It is essential to define the exact cut-off for clinically relevant RAS-mutant allele frequencies in liquid biopsy, that is, the MAF below which a patient is considered to still benefit from anti-EGFR treatment | 1 | 77 | 20 | 3 | 0 | 97 | Consensus (agreement) |
|  |  | 2 | - | - | - | - | - |  |

**Abbreviations:** wt=wild-type; mCRC=metastatic colorectal cancer; CT=chemotherapy; EGFR= epidermal growth factor receptor; MAF=mutant allele fraction.

*Delphi 1^st^ and 2^nd^ rounds response categories: 1=totally agree; 2=basically agree; 3=basically disagree; 4=totally disagree.

†Rate of respondents who voted agree (1 or 2) or disagree (3 or 4) after each Delphi round.

‡Final consensus was defined as: “unanimous” when the response ratein categories 1 and 2 or 3 or 4 was 100%; “consensus” when the response rate in categories 1 and 2 or 3 and 4 was 75-99%; “majority” when the response rate in categories 1 and 2 or 3 and 4 was 60%-74%; “dissent” if the response rate was <60%.
